# A Very Rare Coronary Artery Anomaly: Two Circumflex Arteries Originating From Both Right and Left Coronary Sinuses of Valsalva

**DOI:** 10.14740/cr381w

**Published:** 2015-04-06

**Authors:** Mehmet Demir, Ahmet Tutuncu, Alper Karakus

**Affiliations:** a Bursa Yuksek Ihtisas Education and Research Hospital Cardiology Department, Bursa, Turkey

**Keywords:** Coronary artery anomalies, Twin circumflex arteries, Right sinus of Valsalva

## Abstract

Coronary anomalies are found in less than 1% of diagnostic coronary angiograms. The clinical importance of coronary anomalies varies from insignificant to life-threatening. We report a very rare case of a patient with two circumflex arteries originating from both right and left coronary sinuses of Valsalva.

## Introduction

Coronary artery anomalies have been identified in 0.6-1.5% of coronary angiograms in the general population. The importance of coronary anomalies varies from unimportant to life-threatening [1-3].

## Case Report

**Figure 1:**
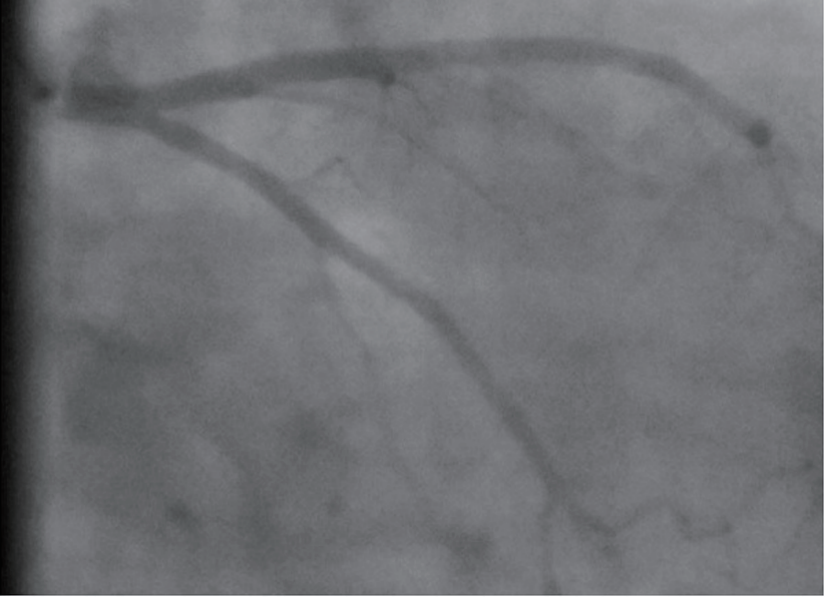
Coronary angiogram in the right caudal view shows the left coronary artery tree with a circumflex artery.

A 52-year-old hypertensive man with a history of chest discomfort was referred to our clinic. His physical examination, echocardiogram, and electrocardiogram reports were all normal. Following physical examination and initial tests, a diagnostic coronary arteriography was performed. Left coronary arteriography revealed a normal left main coronary artery (LMCA) originating from the left sinus of Valsalva. The LMCA was branching into the left anterior descending (LAD) and left circumflex (Cx) arteries (Fig. 1).

**Figure 2:**
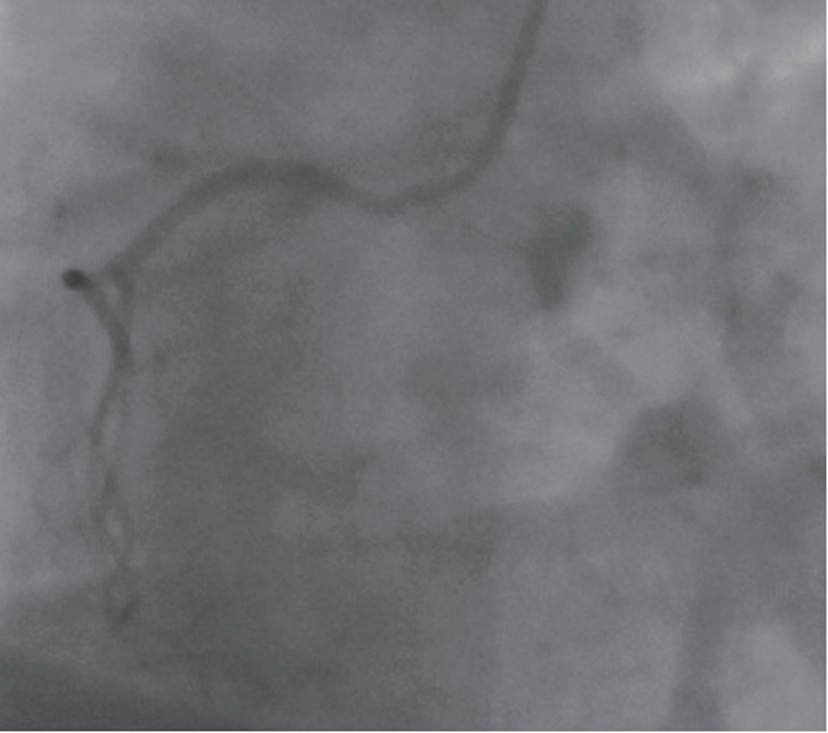
Coronary angiogram in the left anterior oblique cranial view shows the right coronary artery originating from right sinus of Valsalva.

**Figure 3:**
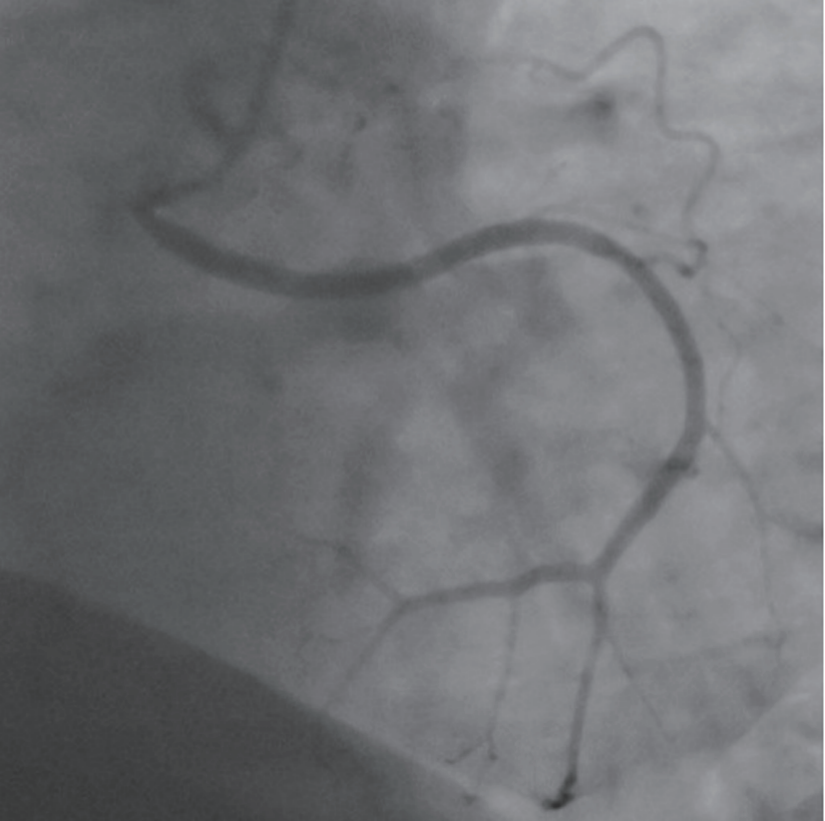
Coronary angiogram in the left anterior oblique cranial view shows the circumflex artery originating from right sinus of Valsalva.

When the right coronary ostium was cannulated, another Cx artery was noticed in addition to a right coronary artery (RCA) (Fig. 2: RCA and Fig. 3: Cx).

The coronary arteries were found to be normal and the patient was discharged without any complication.

## Discussion

The most frequently found anomalies include a Cx artery with a separate ostium from the LAD originating in the left coronary cusp, an origin of the Cx artery taking off from the RCA or arising separately from the right coronary cusp [1-3].

To our knowledge, the case of tricircumflex arteries originating from both right and left coronary sinuses of Valsalva has been previously only two times reported [4-6].

The most important problem in diagnosing double Cx arteries is the separate origin of the two Cx arteries from different ostia on the left or right aortic sinus of Valsalva. Thus, the angiographer must always keep in mind this possibility.

To our knowledge, an aberrant accessory but normal Cx artery has no clinical significance. However, the clinical significance of the anomaly may be important in patients undergoing coronary intervention or cardiac surgery.

A Cx artery arising from the main stem and an anomalous Cx artery from right sinus of Valsalva has been previously reported three times [4-6].

As a fourth we report two circumflex arteries originating from both right and left coronary sinuses of Valsalva in this case.
